# Buyang Huanwu Decoction Targets SIRT1/VEGF Pathway to Promote Angiogenesis After Cerebral Ischemia/Reperfusion Injury

**DOI:** 10.3389/fnins.2018.00911

**Published:** 2018-12-04

**Authors:** Xia-Wei Zheng, Chun-Shuo Shan, Qing-Qing Xu, Yong Wang, Yi-Hua Shi, Yan Wang, Guo-Qing Zheng

**Affiliations:** ^1^Department of Neurology, The Second Affiliated Hospital and Yuying Children’s Hospital of Wenzhou Medical University, Wenzhou, China; ^2^Department of Cardiology, The Second Affiliated Hospital and Yuying Children’s Hospital of Wenzhou Medical University, Wenzhou, China

**Keywords:** Buyang Huanwu Decoction, cerebral ischemia/reperfusion, angiogenesis, silent information regulator 1, vascular endothelial growth factor

## Abstract

**Background:** Ischemia stroke is known as the major cause of morbidity and mortality. Buyang Huanwu Decoction (BHD), a classical traditional Chinese medicine (TCM) formula, has been used to prevent and treat stoke for hundreds of years. The purpose of present study is to investigate the effects of BHD on angiogenesis in rats after cerebral ischemia/reperfusion (I/R) injury targeting Silent information regulator 1 (SIRT1) / Vascular endothelial growth factor (VEGF) pathway.

**Methods:** The cerebral I/R injury model was induced by middle cerebral artery occlusion (MCAO). Adult Sprag-Dawley (SD) rats were randomly divided into five groups: sham group, normal saline (NS) group, BHD group, BHD+EX527 (SIRT1 specific inhibitor) group, and NS+EX527 group. Each group was divided into the subgroups according to 1, 3, 7, or 14 days time-point after cerebral ischemia/reperfusion, respectively. Neurological function score (NFS) was evaluated by the Rogers scale; microvascular density (MVD) in brain tissue around infarction area was observed by immunofluorescence; and the expression of SIRT1 and VEGF was assessed by Western Blot and Quantitative Real-time-PCR.

**Results:** BHD can significantly improve NFS (*P* < 0.05), increase the MVD in the boundary ischemic area (*P* < 0.01) and elevate the expression of protein and mRNA of SIRT1 and VEGF following I/R injury (*P* < 0.01). In contrast, treatment with EX527 reversed the BHD-induced improvements in NFS (*P* < 0.01) and decreased the MVD (*P* < 0.01) and the expression of SIRT1 and VEGF (*P* < 0.05).

**Conclusion:** BHD exerts neuroprotection targeting angiogenesis through the up-regulation of SIRT1/VEGF pathway against cerebral ischemic injury in rats.

## Introduction

Stroke is characterized by acute disturbance of cerebral blood circulation, leading to long-term disability and death worldwide (GBD 2016 Causes of Death et al., 2017). Global health estimates in 2016 from the World Health Organization have shown that stroke caused 5.7 million deaths around the world. In China, the national epidemiological survey of stroke revealed that the prevalence, incidence, and mortality rates of stroke were 1114.8/100 000 people, 246.8 and 114.8/100 000 person-years, respectively (Wang et al., 2017). Stroke not only causes great suffering for patients and family members, but also brings heavy financial burden for individuals and society (Benjamin et al., 2018). The most frequent subtype of all stroke events is ischemic stroke, accounting for 87% (Benjamin et al., 2018). Current treatments of ischemic stroke recommended by guidelines are mainly intravenous thrombolysis of tissue plasminogen activator (tPA) and mechanical thrombectomy (Powers et al., 2018). However, the limitations of tPA for acute ischemic stroke are a narrow time window of 4.5 h after stroke onset and potential risk of fatal intracranial hemorrhage (Fischer et al., 2017). In addition, the successful delivery of mechanical thrombectomy requires specialized stroke center and trained vascular surgeon, which are unavailable at most hospitals (Liebig et al., 2018). Thus, there is an urgent need to explore novel treatment options of stroke.

The concept of the neurovascular unit (NVU) highlights the intimate relationship between the brain and vessels (Iadecola, 2017). Angiogenesis and neurogenesis are considered as the main combined neurovascular responses for the repair of stroke (Arai et al., 2009). The higher density of the new capillaries in the injured area is closely related to a better prognosis and a lower mortality in ischemic stroke patients (Manoonkitiwongsa et al., 2001), suggesting that active angiogenesis may be a promising approach for stroke recovery. Sirtuin 1 (SIRT1), a NAD^+^-dependent class III histone deacetylase, expresses in all cell types. It targets a wide range of transcription factors and consequently, is involved in many physiological and pathological processes as diverse as energy metabolism and myogenesis (Michan and Sinclair, 2007), oxidation-stress (Lee et al., 2018), inflammation (Velagapudi et al., 2017), and apoptosis (Hu T. et al., 2017). Recent studies (Potente et al., 2007; Ota et al., 2010) have demonstrated that SIRT1 is highly expressed in the vasculature during blood vessel growth and regulate the angiogenic activity of endothelial cells. Loss of SIRT1 function blocks inhibits blood vessel development and vascular remodeling. Hu et al. (2016) found that ZYZ-803 regulated angiogenesis through an SIRT1/ Vascular endothelial growth factor (VEGF) /cGMP pathway. In addition, Dong et al. (2008) revealed that resveratrol, one of SIRT1 agonists, significantly elevated levels of protein of MMP-2 and VEGF on focal cerebral ischemic injury in the delayed phase. These evidences suggested that SIRT1/VEGF pathway plays a critical role in angiogenesis during stroke recovery.

Traditional Chinese medicine (TCM) has been applied in the treatment of cerebral ischemia in China for thousands of years (Wang et al., 2011). Buyang Huanwu Decoction (BHD) is a classical TCM formula, recorded in *Corrections on the Errors of Medical Works* written by Qingren Wang in Qing Dynasty. The formula consists of seven herbs, including Radix Astragali seu Hedysari (Huang qi), Radix Angelicae Sinensis (Dang gui wei), Radix Paeoniae Rubra (Chi shao), Lumbricus (Di long), Semen Persicae (Tao ren), Flos Carthami (Hong hua), and Rhizoma Ligustici Chuanxiong (Chuan xiong), in the ratio of 120:6:4.5:3:3:3:3. Our previous clinical and pre-clinical systematic reviews (Hao et al., 2012; Wei et al., 2013) indicated that BHD can improve neurological deficit and be generally safe in patients with acute ischemic stroke, and possess substantial neuroprotective effects in experimental stroke. Angiogenesis is directly linked to neurogenesis after cerebral ischemic injury. BHD can stimulate the processes of adult angiogenesis and neurogenesis in ischemic brain (Shen et al., 2014; Zhang et al., 2016). However, the mechanisms remain unclear. Thus, we aim to investigate the effects of BHD on angiogenesis in rats after cerebral ischemia/reperfusion (I/R) injury through SIRT1/VEGF pathway.

## Materials and Methods

### Ethics Statement

All animals from Shanghai Laboratory Animal Center (License number: SCXK (Hu), 2010-0002) were used in our experiments. The protocol was approved by the local ethic committee of the Wenzhou Medical University. Procedures involving animals and their care were conducted in accordance with the National Institutes of Health Guide for the Care and Use of Laboratory Animals (Publication No. 85-23). All the animals were sacrificed by anesthesia at the end of the experiment. The utmost possible efforts were made to reduce the number of animals used and minimize animal suffering.

### Animals and Groups

Adult male Sprag-Dawley (SD) rats weighing 250–280 g were housed in a climate-controlled room (12 h light/dark cycle with humidity of 50% and temperature of 21–25°C) and were allowed free access to food and water.

Healthy SD rats were randomly divided into five groups: sham group, normal saline (NS) group, BHD group, BHD+EX527 (SIRT1 specific inhibitor) group, and NS+EX527 group. The sham group, NS group, and BHD group were further broken down into four sub-groups according to the time points of 1, 3, 7, and 14 days after I/R injury, respectively. There was only one group (14 days after I/R) in BHD+EX527 group and NS+EX527 group, respectively. There are 12 rats in the sub-group of each time point.

### Drug Administration

Buyang Huanwu Decoction is a granule made up of Radix Astragali seu Hedysari, Radix Angelicae Sinensis, Radix Paeoniae Rubra, Lumbricus, Semen Persicae, Flos Carthami, Rhizoma Ligustici Chuanxiong according to the ratio of 120 : 6 : 4.5 : 3 : 3 : 3 : 3 (Manufacturer: Huarun three nine Pharmaceutical Company Ltd. Approval number: country medicine accurate character Z44020711) and dissolved in distilled water at 100°C. BHD at dose of 14.25 g/kg was administered intragastrically to the rats in BHD group and BHD+EX527 goup according to the human equivalent dose. Same volume of NS was administered intragastrically to the rats in sham group, NS group and NS+EX527 group instead. Administration of BHD or NS was performed once a day beginning at 3 days before the operation until sacrificing the rats. Intracerebroventricular injection of EX527 at the dose of 10 μg was performed to the rats in BHD+EX527 groups and NS+EX527 group, every 2 days from 7 days before the operation until the rats were sacrificed.

### Cerebral Ischemia/Reperfusion (I/R) Injury

I/R was established according to the modified Zea Longa method (1989). Briefly, after being anesthetized with 4% chloral hydrate (3 ml/kg) intraperitoneally and fixed in a supine position, the left common carotid artery (CCA), internal carotid artery (ICA), and external carotid artery (ECA) of rats were isolated through the midline incision of neck. A 0.26 mm diameter monofilament nylon suture with rounded tip (Beijing Cinontech Co., Ltd., Beijing, China) was inserted into ECA and gently advanced into the ICA in order to block the origin of the middle cerebral artery (MCA). Rats in sham group underwent identical procedure except that the suture only stays in ICA. After 2 h occlusion, reperfusion was achieved by slowly removing the suture. The rats awaken from anesthesia were returned to cages and free to food and water.

### Neurological Function Score (NFS)

Neurological function score were examined at 1, 3, 7, and 14 days after I/R by an investigator who was blind to the experiment grouping using the Rogers scale (Gutiérrez-Fernández et al., 2011; Zhou et al., 2015) as follows: 0, no deficit; 1, failure to extend left forepaw; 2, decreased grip of the left forelimb when the tail is pulled; 3, spontaneous movement in all directions, contralateral circling if pulled; 4, circling or walking to the left; 5, movement only when stimulated; 6, unresponsive to stimulation; and 7, dead. Rats with a score of 0, 6, or 7 after 6 h of reperfusion were excluded from the study.

### Immunofluorescence Staining

Rats were anesthetized at 1, 3, 7, and 14 days after I/R, and the brains were removed after perfusion with NS and 4% paraformaldehyde. After gradient elution with sucrose, the brains were quickly frozen and cut into 8 μm coronal thick sections. The sections were permeabilized with 0.5% Triton X-100 for 5 min, were blocked with 10% donkey serum for 1 h, and then incubated with mouse anti-CD31 antibody (1:100, Abcam, United States) overnight at 4°C. Thereafter, sections were briefly washed with PBST and incubated with donkey anti-mouse secondary antibody (1:100, Cell Signal, United States) for 1 h at 37°C. After counterstaining with 4,6-diamidino-2-phenylindole (DAPI; Beyotime, Shanghai, China) and coversliping with anti-fade mounting medium (Beyotime, Shanghai, China), the sections were observed and photographed under fluorescent microscopy and then analyzed with Image-Pro Plus 6.0 software.

### Western Blot Analysis

Brain tissues from hippocampus were lysed in RIPA buffer (Beyotime, Shanghai, China). The supernatants were collected and then quantitated for protein determination using a BCA Protein Assay kit (Beyotime, Shanghai, China). Denatured protein samples were separated on 8% SDS-polyacrylamide gels (SDS-PAGE), and transferred to polyvinyl difluoridine (PVDF) membranes. The membrane was blocking with 5% non-fat dry milk for 2 h and incubated with rabbit anti- SIRT1 antibody (diluted 1:1000; Cell Signal, United States), rabbit anti- VEGF antibody (diluted 1:1000; Cell Signal, United States) or rabbit anti- β-actin antibody (diluted 1:5000; Cell Signal, United States) overnight at 4°C. Then, the membrane was incubated with goat anti-rabbit IgG secondary antibody (diluted 1:3000; Cell Signal, United States) for 2 h at room temperature. The protein bands were visualized using enhanced chemiluminescence (ECL) and were quantified by scanning densitometry using Image J software.

### Real-Time Quantitative Reverse Transcription Polymerase Chain Reaction (RT-qPCR)

Total RNA was isolated using Trizol reagent (Invitrogen, United States). RNA samples from each group were reverse transcribed into cDNA using PrimeScriptTM RT reagent Kit (TAKARA, Japan). Quantitative RT-qPCR was performed on a Light Cycler thermal cycler system (Bio-Rad, United States) using SYBR^®^Premix Ex Taq^TM^II (TAKARA, Japan) and gene-specific primers. Gene-specific primers used as followed: SIRT1: forward, 5′- CATACTCGCCACCTAACCTAT -3′ and revised, 5′- AACCTCTGCCTCATCTACATTT -3′, at a fragment length of 93 bp; VEGFA: forward, 5′- CCTCTCCCTACCCCACTTCCT -3′ and revised, 5′- CACTTTCTCTTTTCTCTGCCTCCAT -3′, at a fragment length of 196 bp; β-actin: forward, 5′- CCGTAAAGACCTCTATGCCAACA -3′ and revised, 5′- CTAGGAGCCAGGGCAGTAATCTC -3′, at a fragment length of 102 bp.

### Statistical Analysis

All statistical analyses were performed using SPSS software (version 20.0) and values are expressed as means ± standard deviation (mean ± SD). Differences between multiple groups were analyzed by One-way analysis of variance (ANOVA) and differences between two groups were analyzed using the *t*-test. Significant difference was considered when *P* < 0.05.

## Results

### Effect of BHD on Neurological Deficits

**FIGURE 1:**
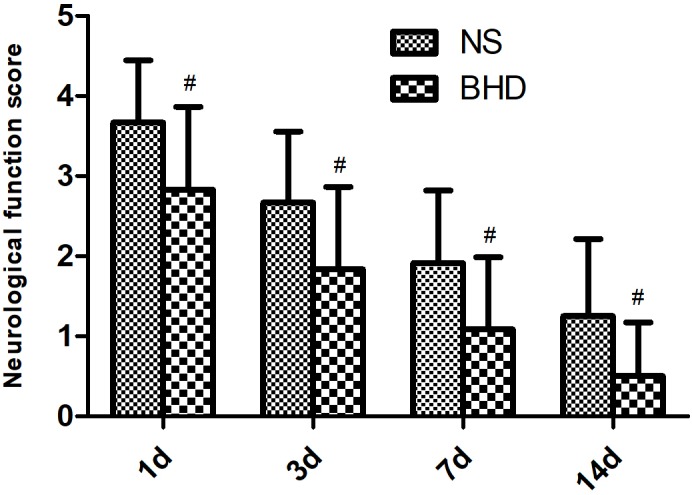
The neurological function score (NFS) in normal saline (NS) group and Buyang Huanwu Decoction (BHD) group at 1, 3, 7, and 14 days after I/R in rats (mean ± SD, *n* = 12). ^#^*P* < 0.05, compared with NS group.

No neurological deficits were detected among the sham group. In NS group, NFS significantly decreased at 3 days after cerebral I/R when compared with that at 1 day (*P* < 0.05); NFS significantly decreased at 7 days when compared with 3 days (*P* < 0.05); NFS significantly decreased at 14 days when compared with 7 days (*P* < 0.05). In BHD group, NFS significantly decreased at 3 days when compared with 1 day (*P* < 0.05); NFS significantly decreased at 7 days when compared with 3 days (*P* < 0.05); NFS significantly decreased at 14 days when compared with 7 days (*P* < 0.05). Compared with NS group, NFS significantly decreased in BHD group at 1, 3, 7, and 14 days (*P* < 0.05) (Figure 1).

### Effect of BHD on Microvascular Density (MVD)

**FIGURE 2:**
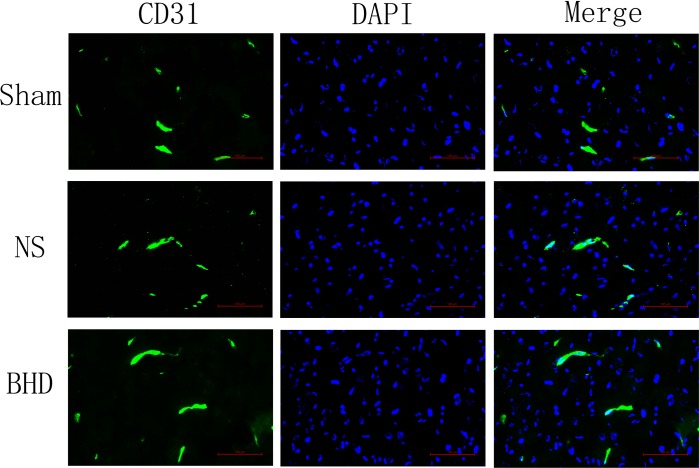
Immunofluorescence staining of CD31-positive microvascular density (MVD) around the infarction in cortex at 1 day after I/R (mean ± SD, *n* = 6). Scale bar: 100 μm.

**FIGURE 3:**
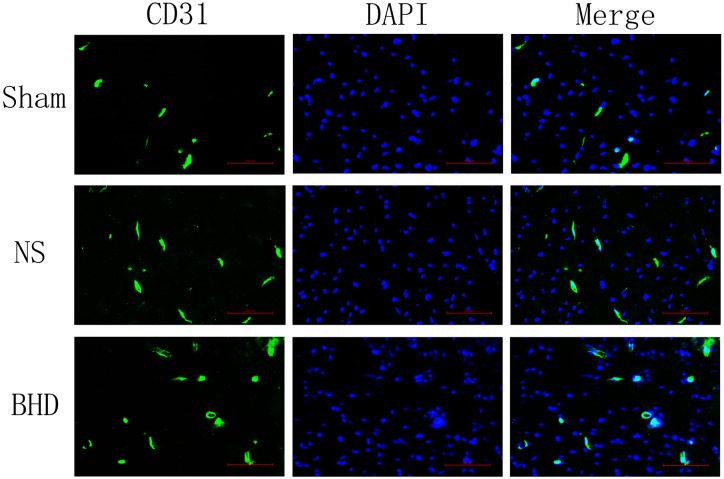
Immunofluorescence staining of CD31-positive MVD around the infarction in cortex at 3 days after I/R (mean ± SD, *n* = 6). Scale bar: 100 μm.

**FIGURE 4:**
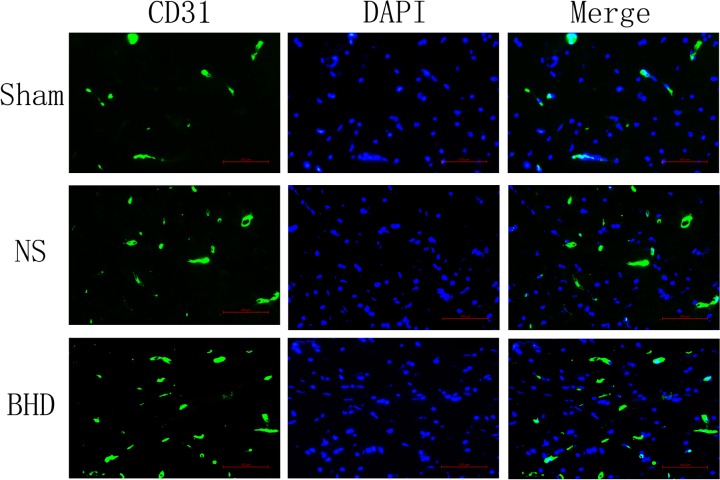
Immunofluorescence staining of CD31-positive MVD around the infarction in cortex at 7 days after I/R (mean ± SD, *n* = 6). Scale bar: 100 μm.

**FIGURE 5:**
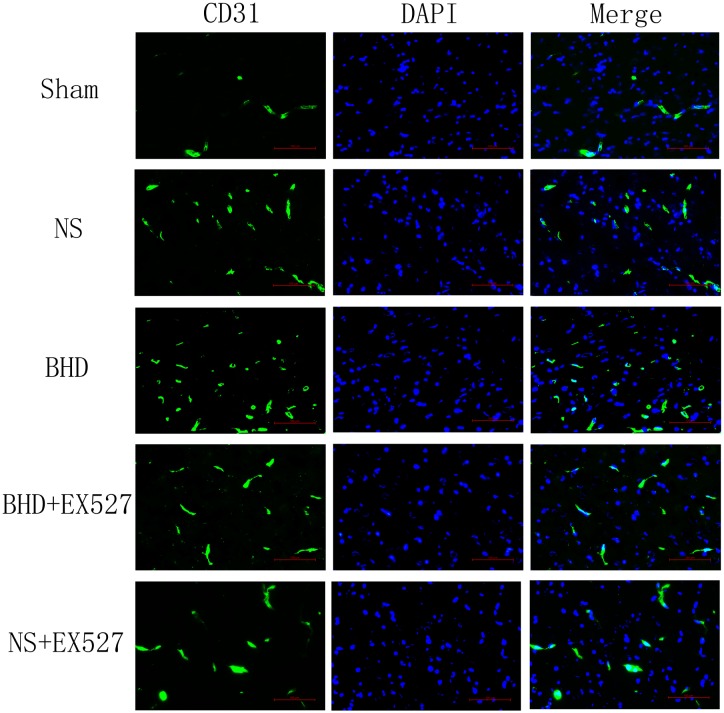
Immunofluorescence staining of CD31-positive MVD around the infarction in cortex at 14 days after I/R (mean ± SD, *n* = 6). Scale bar: 100 μm.

**FIGURE 6:**
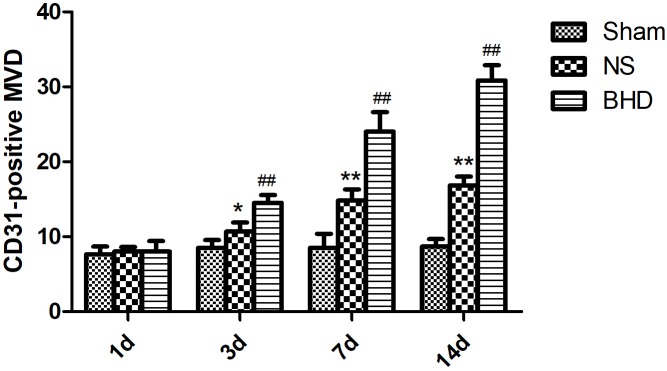
Quantitative analysis for the results of immunofluorescence staining of CD3-positive MVD around the infarction in cortex in Sham, NS, and BHD group at 1, 3, 7, and 14 days after I/R (mean ± SD, *n* = 6). ^∗^*P* < 0.05, ^∗∗^*P* < 0.01, compared with Sham group; ^##^*P* < 0.01, compared with NS group.

Immunofluorescence staining showed that MVD had no significant difference in sham group among the time point of 1, 3, 7, and 14 days. In NS group, MVD significantly increased at 3 days after I/R when compared with 1 day (*P* < 0.01); MVD significantly increased at 7 days when compared with 3 days (*P* < 0.01); MVD significantly increased at 14 days when compared with 7 days (*P* < 0.01). In BHD group, MVD significantly increased at 3 days when compared with 1 day (*P* < 0.01); MVD significantly increased at 7 days when compared with 3 days (*P* < 0.01); MVD significantly increased at 14 days when compared with 7 days (*P* < 0.01). Compared with sham group, MVD significantly increased at 3, 7, and 14 days (*P* < 0.05) in NS group but had no significant difference at 1 day. Compared with NS group, MVD significantly increased at 3, 7, and 14 days in BHD group (*P* < 0.01) but had no significant difference at 1 day (Figures 2–6).

### Effect of BHD on the Expression of SIRT1 in the Hippocampus

**FIGURE 7:**
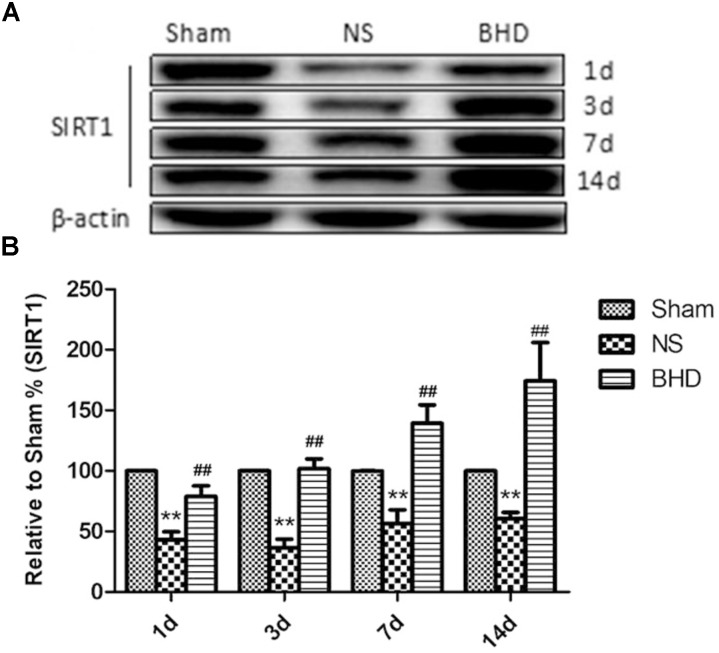
Western blot analysis of the expression of SIRT1 in hippocampal in Sham, NS, and BHD group at 1, 3, 7, and 14 days after I/R (mean ± SD, *n* = 6). **(A)**, Expression of SIRT1 in focal rat brain coronal frozen sections. **(B)**, quantitative analysis for the western blot results of SIRT1 at 1, 3, 7, and 14 days, respectively. ^∗∗^*p* < 0.001, compared with Sham group. ^##^*P* < 0.01, compared with NS group.

**FIGURE 8:**
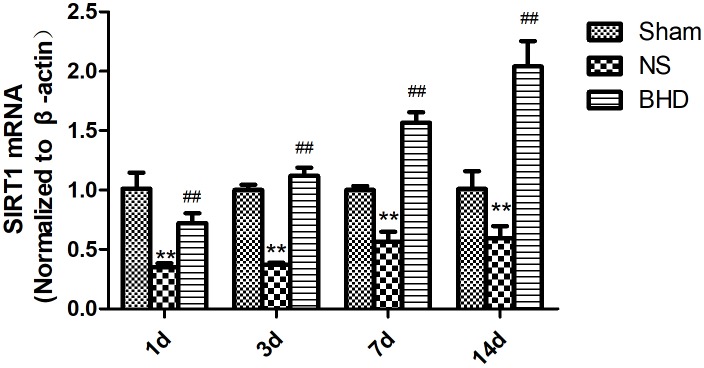
The SIRT1 mRNA expression in hippocampal in Sham, NS, and BHD group at 1, 3, 7, and 14 days after I/R (mean ± SD, *n* = 6). ^∗∗^*p* < 0.01, compared with Sham group. ^##^*P* < 0.01, compared with NS group.

Western Blot and RT-q PCR showed that the expression of SIRT1 had no significant difference in sham group among the time point of 1, 3, 7, and 14 days. In NS group, the expression of SIRT1 protein and mRNA significantly increased at 7 days after I/R when compared with 3 days (*P* < 0.01); the expression of SIRT1 protein and mRNA had no significant difference between 1 and 3 days, and 7 and 14 days. In BHD group, the expression of SIRT1 protein and mRNA significantly increased at 3 days when compared with 1 day (*P* < 0.01); the expression of SIRT1 protein and mRNA significantly increased at 7 days when compared with 3 days (*P* < 0.01); the expression of SIRT1 protein and mRNA significantly increased at 14 days when compared with 7 days (*P* < 0.05). Compared with Sham group, SIRT1 protein and mRNA significantly decreased in NS group at 1, 3, 7, and 14 days (*P* < 0.01). Compared with NS group, the expression of SIRT1 protein and mRNA significantly increased in BHD group at 1, 3, 7, and 14 days (*P* < 0.01) (Figures 7, 8).

### Effect of BHD on the Expression of VEGF in the Hippocampus

**FIGURE 9:**
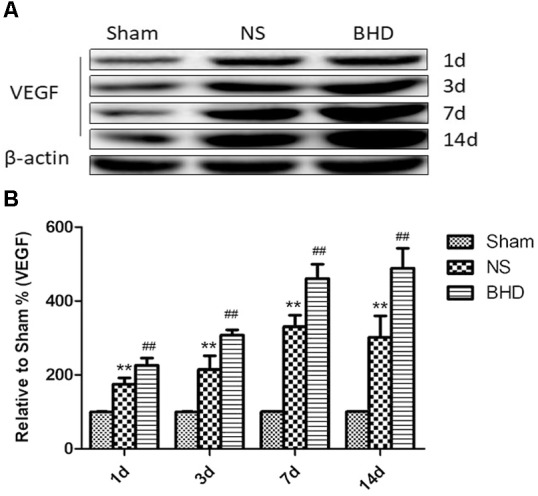
Western blot analysis of the expression of Vascular endothelial growth factor (VEGF) in hippocampal in Sham, NS, and BHD group at 1, 3, 7, and 14 days after I/R (mean ± SD, *n* = 6). **(A)** Expression of VEGF in focal rat brain coronal frozen sections. **(B)** quantitative analysis for the western blot results of VEGF at 1, 3, 7, and 14 days, respectively. ^∗∗^*p* < 0.01, compared with Sham group. ^##^*P* < 0.01, compared with NS group.

**FIGURE 10:**
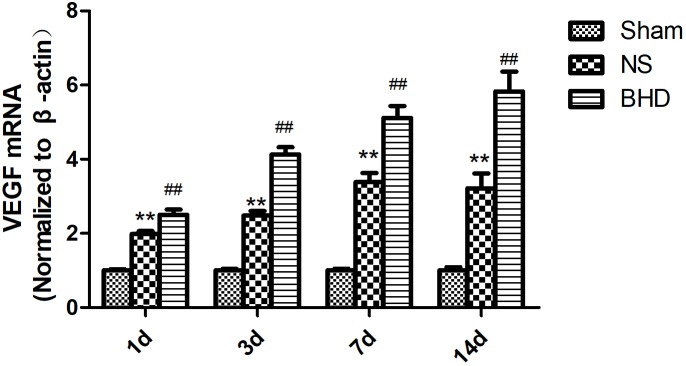
The VEGF mRNA expression in hippocampal in Sham, NS, and BHD group at 1, 3, 7, and 14 days after I/R (mean ± SD, *n* = 6). ^∗∗^*p* < 0.01, compared with Sham group. ^##^*P* < 0.01, compared with NS group.

Western Blot and RT-q PCR showed that the expression of VEGF had no significant difference in sham group among the time point of 1, 3, 7, and 14 days. In NS group, the expression of VEGF protein and mRNA significantly increased at 3 days when compared with 1 day (*P* < 0.05); the expression of VEGF protein and mRNA significantly increased at 7 days when compared with 3 days (*P* < 0.01); the expression of VEGF protein and mRNA had no significant difference between 14 and 7 days. In BHD group, the expression of VEGF protein and mRNA significantly increased at 3 days when compared with 1 day (*P* < 0.01);the expression of VEGF protein and mRNA significantly increased at 7 days when compared with 3 days (*P* < 0.01);the expression of VEGF protein and mRNA significantly increased at 14 days when compared with 7 days (*P* < 0.05). Compared with sham group, VEGF protein and mRNA significantly increased in NS group at 1, 3, 7, and 14 days (*P* < 0.01). Compared with NS group, the expression of VEGF protein and mRNA significantly increased in BHD group at 1, 3, 7, and 14 days (*P* < 0.01) (Figures 9, 10).

### EX527 Inhibits the Improvement of Neurological Deficit Scores Induced by BHD

**FIGURE 11:**
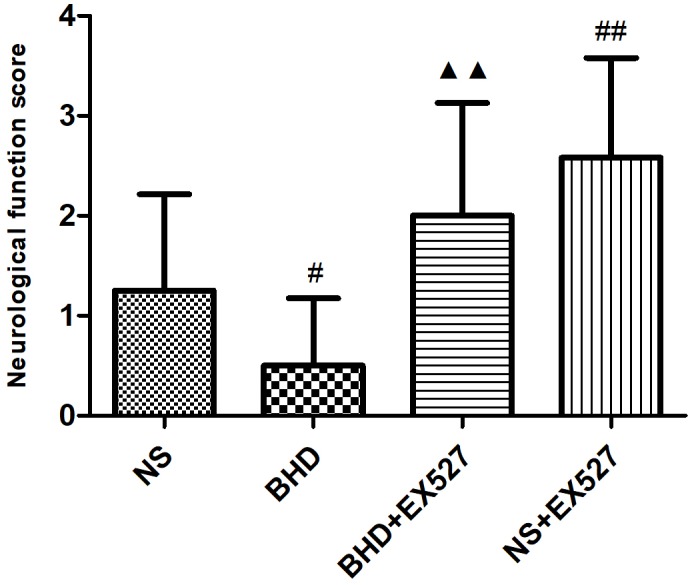
The NFS in NS, BHD, BHD+EX527, and NS+EX527 group at 14 days after I/R in rats (mean ± SD, *n* = 12). ^#^*P* < 0.05, compared with NS group. ^#^*P* < 0.05, ^##^*P* < 0.01, compared with NS group. ^▲▲^*p* < 0.01, compared with BHD group.

After cerebral I/R injury, NFS significantly increased in BHD+EX527 group when compared with BHD group at 14 days (*P* < 0.01). NFS significantly increased in NS+EX527 group when compared with NS group at 14 days (*P* < 0.01). Compared with the NS+EX527 group, BHD+EX527 group had lower NFS but without statistically significant difference at 14 days (Figure 11).

### EX527 Inhibits the Increase of MVD Induced by BHD

**FIGURE 12:**
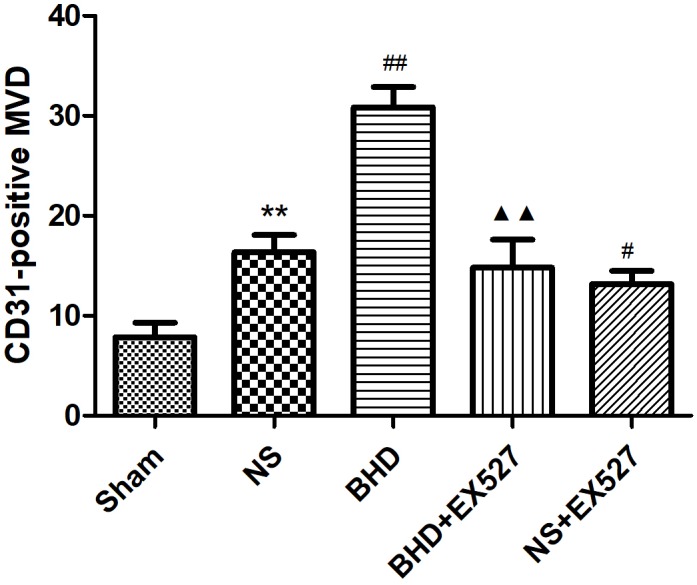
Quantitative analysis for the results of immunofluorescence staining of CD3-positive MVD around the infarction in cortex in Sham, NS, BHD, BHD+EX527, and NS+EX527 group at 14 days after I/R (mean ± SD, *n* = 6). ^∗^*P* < 0.05, ^∗∗^*P* < 0.01, compared with Sham group; ^##^*P* < 0.01, compared with NS group.

After cerebral I/R injury, MVD significantly decreased in BHD+EX527 group when compared with BHD group at 14 days (*P* < 0.01). MVD significantly decreased in NS+EX527 group when compared with NS group at 14 days (*P* < 0.05). MVD had no significant difference between BHD+EX527 group and NS+EX527 group at 14 days (Figure 12).

### EX527 Inhibits the Up-Regulation of SIRT1 and VEGF Induced by BHD

**FIGURE 13:**
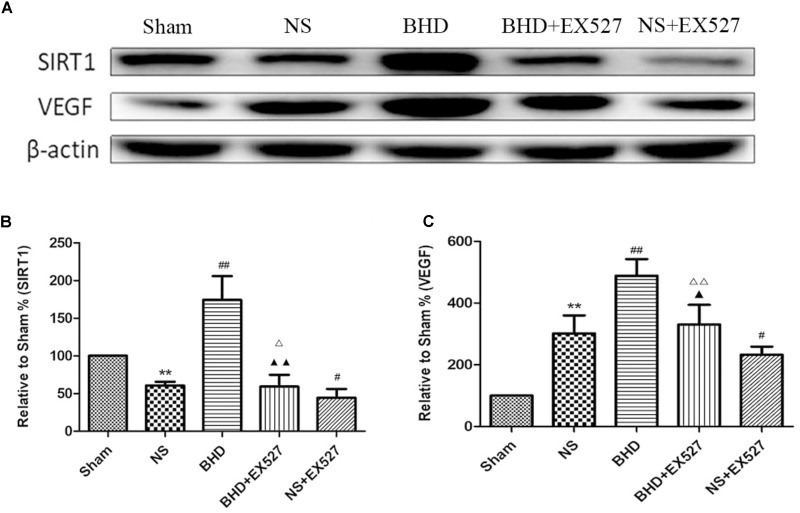
Western blot analysis of the expression of SIRT1 and VEGF in hippocampal in Sham, NS, BHD, BHD+EX527, and NS+EX527 group at 14 days after I/R (mean ± SD, *n* = 6). **(A)** Expression of SIRT1 and VEGF in focal rat brain coronal frozen sections. **(B)** quantitative analysis for the western blot results of SIRT1 at 14 days. **(C)** quantitative analysis for the western blot results of VEGF at 14d. ^∗^*P* < 0.05, ^∗∗^*P* < 0.01, compared with Sham group; ^##^*P* < 0.01, compared with NS group; ^▲^*P* < 0.05, ^▲▲^*P* < 0.01, compared with BHD group; ^Δ^ *P* < 0.05, ^Δ Δ^ *P* < 0.01, compared with NS+EX527 group.

Western Blot showed that compared with BHD group, the expression of SIRT1 and VEGF protein in hippocampus was significantly decreased in BHD+EX527 group (*P* < 0.05). Compared with the NS group, there was no significant difference in the SIRT1 and VEGF protein expression in the BHD+EX527 group. Compared with the NS group, the expression of SIRT1 and VEGF protein in hippocampus was higher than that in the NS+EX527 group (*P* < 0.05). Compared with NS+EX527 group, the expression of SIRT1 and VEGF protein in hippocampus was significantly increased in BHD+EX527 group (*P* < 0.05). The increase of SIRT1 and VEGF protein expression between NS+EX527 group and BHD+EX527 group was less than that between NS group and BHD group (*P* < 0.05) (Figure 13).

**FIGURE 14:**
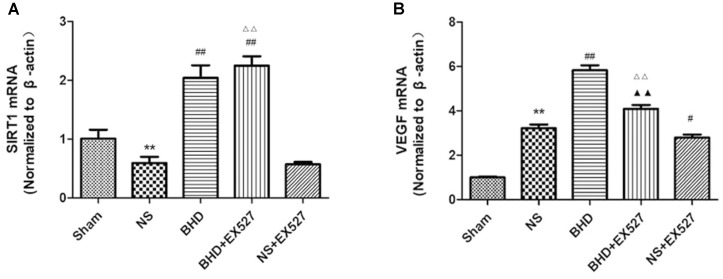
The SIRT1 and VEGF mRNA expression in hippocampal in Sham, NS, BHD, BHD+EX527, and NS+EX527 group at 14 days after I/R (mean ± SD, *n* = 6). **(A)** The SIRT1 mRNA expression. **(B)** The VEGF mRNA expression. ^∗∗^*P* < 0.01, compared with Sham group; ^#^*P* < 0.05, ^##^*P* < 0.01, compared with NS group; ^▲▲^*P* < 0.01, compared with BHD group; ^Δ Δ^ *P* < 0.01, compared with NS+EX527 group.

RT-PCR showed that compared with BHD group, the expression of VEGF mRNA in hippocampus was significantly decreased in BHD+EX527 group (*P* < 0.01). There was no significant difference in the expression of SIRT1 mRNA between BHD group and BHD+EX527 group. Compared with NS group, the SIRT1 mRNA expression was higher than BHD+EX527 group (*P* < 0.01), while there was no significant difference on the VEGF mRNA between the two groups. Compared with the NS group, the VEGF mRNA expression was significantly decreased in NS+EX527 group (*P* < 0.05), while there was no significant difference on the SIRT1 mRNA between the two groups. Compared with NS+EX527 group, the expressions of SIRT1 and VEGF mRNA in hippocampus were significantly increased in BHD+EX527 group (*P* < 0.01). There was no significant difference in SIRT1 mRNA between NS+EX527 group and BHD+EX527 group. The expression of SIRT1 mRNA between NS group and BHD group had no significant difference. The expression of VEGF mRNA between NS+EX527 group and BHD+EX527 group was significantly less than that between NS group and BHD group (*P* < 0.05) (Figure 14).

## Discussion

This study demonstrated that BHD can significantly improve neurological deficit, increase the MVD in the boundary ischemic area and elevate the expression of SIRT1 and VEGF following I/R injury. In contrast, EX527, a specific inhibitor of SIRT1 inhibited the improvement of neurological function and angiogenesis induced by BHD in rats with cerebral I/R. Thus, BHD exerted the neuroprotective effect through the active angiogenesis that targets SIRT1/VEGF pathway in rat’s brain I/R injury.

Silent information regulator 1, an NAD^+^-dependent class III histone deacetylase, is involved in different biological processes such as oxidative stress, autophagy, neuroprotection and mitochondrial function (Horio et al., 2011). SIRT1 was abundantly expressed in the brain, especially in the piriform cortex, hippocampus, hypothalamus, the medial habenular nucleus, and cerebellum (Ramadori et al., 2008). SIRT1 plays an essential role in cerebrovascular diseases. The neuroprotective effects of SIRT1 in stroke have been reported. Compared with the wild-type mice, SIRT1-knockout mice had larger infarct volumes following permanent focal ischemia (Hernández-Jiménez et al., 2013), and in contrast, mice with SIRT1 overexpression showed less hippocampal damage after bilateral common carotid artery occlusion (BCAO) (Hellberg et al., 2010). Furthermore, EX527 is a specific inhibitor of SIRT1 (Qin et al., 2018). In the present study, MCAO rats were treated with BHD in the presence or absence of EX527. Consistent with above findings, in our study, inhibition of SIRT1 by EX527 increased NFS of NS group, verifying the protective effects of SIRT1 in I/R injury. Moreover, inhibition of SIRT1 abolished the protective effect of BHD in neurologic impairment, suggesting that BHD exerted neuropotective effects against I/R injury via up-regulating the expressions of SIRT1. SIRT1 is also a key regulator of vascular endothelial homeostasis controlling angiogenesis and vessel function (Potente and Dimmeler, 2008). Ota et al. (2010) reported that SIRT1/eNOS axis can be a potential target against vascular senescence, dysfunction and atherosclerosis. Potente et al. (2007) showed that knockdown of SIRT1 results in defective blood vessel formation and blunts ischemia-induced neovascularization. MVD is considered to be a valuable parameter for quantitatively assessing angiogenesis (Zhang et al., 2018). Several vascular endothelial cell markers, such as CD31 (Chen et al., 2018), CD34 (Hu G.J. et al., 2017), and FVIII antigens (Tang et al., 2018), which stain both mature pre-existing vessels and newly formed blood vessels, have been utilized for the detection of MVD. In our study, MVD obtained by immunofluorescence staining of CD31. MVD significantly increased at 3 days after I/R. BHD could further upregulate MVD at 3, 7, and 14 days after reperfusion, indirectly indicating the angiogenesis effects of BHD. In contrast, inhibition of SIRT1 by EX527 significantly decreased MVD in I/R rats, verifying the angiogenesis effects of SIRT1 in I/R rats. Moreover, inhibition of SIRT1 abolished the angiogenesis effect of BHD, suggesting that BHD promoted angiogenesis in I/R injury via up-regulating the expressions of SIRT1.

Vascular endothelial growth factor is produced and secreted by many neurovascular cells in brain (Yin et al., 2015) and is considered to be a central mediator in post-ischemic angiogenesis. In the experimental cerebral ischemic models, VEGF was upregulated at the border of the infarction commencing 3 h after MCAO, and continued 3–7 days following stroke (Plate et al., 1999). Mounting evidence has showed that VEGF combines with its receptors and triggers multiple downstream signals, thereby promoting angiogenesis (Greenberg and Jin, 2005; Beck and Plate, 2009). Several studies have explored the associations between SIRT1 and VEGF in angiogenesis. An *in vitro* study of Kudo et al. (2018) showed that mRNA expression of SIRT1 in Human umbilical vein endothelial cells (HUVECs) peaked after 6 h of incubation with a SIRT1 activator, resveratrol. However, VEGF mRNA showed delayed expression, 4 h later than SIRT1, suggesting that SIRT1-mediated stimulation of expression. Meanwhile, inhibitor experiment exhibited only control levels of VEGF in the absence of SIRT1 expression. In the model of cerebral ischemia in mice (Dong et al., 2008), resveratrol was reported to significantly up-regulate the expression of VEGF and MMP-2, thereby attenuating ischemic brain damage in the delayed phase. In our study, the expression of VEGF started to increase significantly at 1 day after reperfusion. BHD could further upregulate the expression of VEGF at 1, 3, 7, and 14 days after reperfusion. In contrast, inhibition of SIRT1 by EX527 abolished the level of VEGF mediated by BHD, suggesting BHD promoted the expression of VEGF in I/R injury via up-regulating the expressions of SIRT1. Interestingly, VEGF was reported to cause vascular permeability and edema by uncoupling endothelial cell–cell junctions, resulting in extensive injury to ischemic tissues after stroke (Weis and Cheresh, 2005). However, blood brain barrier is a site with multi-target, not only VEGF. BHD prescription is a complicated multi-component and multi-pathway system. Several evidences have showed that BHD could attenuate BBB disruption. Chen et al. (2015) reported that BHD significantly preserved the BBB by increasing Occludin and CaMKII and reducing apoptosis in infarct areas. Dou et al. (2018) suggested that the BBB protection of BHD may involve the inhibition of NF-kB-associated CXCL10-CXCR3-mediated chemotaxis, and thus contribute to the prevention of NK cell infiltration in the ischemic brain. Further research is needed to explore the VEGF paradox in different stages of stroke.

The present study still has several limitations. We explored BHD targets the SIRT1/VEGF signal pathway in angiogenesis after stroke. However, downstream molecules of VEGF in BHD-induced angiogenesis needs further to be verified. In addition, BHD prescription is a complicated multi-component and multi-pathway system. To date, hundreds of constituents have been isolated from the BHD such as polysaccharides, astragalosides, and isoflavonoids in Radix Astragali seu Hedysari (Chu et al., 2010), as well as phthalides and phenolic acids in Radix Angelicae Sinensis and Rhizoma Ligustici Chuanxiong (Liu et al., 2010; Ran et al., 2011). Accordingly, specific constituents are responsible for the neuroprotective and angiogenesis effects of BHD that need further investigation.

## Conclusion

The present study demonstrates that BHD can improve NFS, increase MVD and promote angiogenesis of I/R in rats. The neuroprotection effects of BHD against cerebral ischemic injury target angiogenesis through up-regulation of SIRT1/VEGF pathway.

## Author Contributions

X-WZ, G-QZ, and YaW designed the study. X-WZ, C-SS, Q-QX, YoW, and Y-HS performed the experiments and analyzed the data. X-WZ and C-SS supervised the study and wrote the paper. All authors participated to the final approval of the version to be published.

## Conflict of Interest Statement

The authors declare that the research was conducted in the absence of any commercial or financial relationships that could be construed as a potential conflict of interest.
